# Relations between the set-complexity and the structure of graphs and their sub-graphs

**DOI:** 10.1186/1687-4153-2012-13

**Published:** 2012-09-21

**Authors:** Tomasz M Ignac, Nikita A Sakhanenko, David J Galas

**Affiliations:** 1 Institute for Systems Biology, 401 N. Terry Avenue, Seattle, WA 98109, USA; 2 Luxembourg Centre for Systems Biomedicine, University of Luxembourg, Campus Belval, 7, Avenue des Hauts-Fourneaux, L-4362 Esch-sur-Alzette, Luxembourg

**Keywords:** Set-complexity, Biological networks, Modularity, Modular graphs, Bipartite graphs, Multi-partite graphs

## Abstract

We describe some new conceptual tools for the rigorous, mathematical description of the “set-complexity” of graphs. This set-complexity has been shown previously to be a useful measure for analyzing some biological networks, and in discussing biological information in a quantitative fashion. The advances described here allow us to define some significant relationships between the set-complexity measure and the structure of graphs, and of their component sub-graphs. We show here that modular graph structures tend to maximize the set-complexity of graphs. We point out the relationship between modularity and redundancy, and discuss the significance of set-complexity in this regard. We specifically discuss the relationship between complexity and entropy in the case of complete-bipartite graphs, and present a new method for constructing highly complex, binary graphs. These results can be extended to the case of ternary graphs, and to other multi-edge graphs, which are fundamentally more relevant to biological structures and systems. Finally, our results lead us to an approach for extracting high complexity modular graphs from large, noisy graphs with low information content. We illustrate this approach with two examples.

## Introduction

Most physical, communications, social, and biological networks are usefully represented as graphs, with varying levels of complexity. The topology and the statistical structures of these graphs are central to understanding the functional properties of these systems. Our primary concern here is the representation and properties of biological networks, as reflected in the graphs used to represent these complex systems. The application of our results, however, is significantly broader. Previous attempts to elucidate the fundamental concept of biological information have led to a proposed, general measure of complexity, or information content, based on Kolmogorov complexity [1,2], that resolves some of the perplexing paradoxes of biologically relevant meaning that arise in definitions of information and complexity [1]. We used this approach successfully in analyzing the information in gene interaction networks of yeast [3,4]. It was shown that the most informative networks are those with the highest set-complexity (a detailed discussion about applications of the set-complexity to biology and related problems can be found in the cited articles). The properties of our measure, which we call “set-complexity”, are expected to be fruitful in describing a large class of problems in biology. It is clear, however, that we need more mathematical understanding of the properties of this complexity measure, and we have therefore focused initially on the set-complexity of graphs, and begun by analyzing the mathematical properties of relatively simple structures.

The results here extend our previous results and increase understanding of the structure of graphs and sub-graphs with the highest set-complexity. We have previously suggested, for example, that highly complex graphs have a more modular architecture than others [4]. The aim of this article is twofold. First, we aim to provide a mathematical foundation for this suggestion, the relation between the set-complexity and the graph structure. Second, we show that this research has practical uses. To accomplish the first goal we develop a formalism that allows us to analyze the set-complexity in a rigorous fashion and capture some of its essential properties. Our approach uses stochastic methods to analyze graphs by defining specific random variables describing interactions between nodes in a graph. Information-theoretical features of the variables defined are then used to investigate the set-complexity, *Ψ*, measure. To accomplish the second goal, we present two examples illustrating how the set-complexity theory can be used to identify specific sub-graphs with modular properties. Note that the theoretical formalism of this article extends the ideas from our previous article [5] that presented a technical background of set-complexity and its computation as well as initial analysis of complexity of some graphs. Article [5] does not touch the application of this formalism in finding modular structure from real-world networks, which is a major goal of this article.

The article is structured as follows. First, we describe basic definitions and notation, and present the relation between the complexity and the entropy for complete bipartite graphs (CBG), an important class of binary graph for this analysis. We then describe a method for constructing highly complex binary graphs and provide two examples which show how to use the set-complexity to analyze information content of a graph and its sub-graphs. We conclude the article by discussing results, open questions and plans for future work.

## Preliminaries

Let *G*=(*V*,*E*) denote a graph, where *V* stands for the set of vertices and *E* the set of edges. The number of nodes in a graph is denoted by *N*, i.e., *V*={1,…,*N*}. Existence of an edge between nodes *i* and *j* is denoted by (*i*,*j*)∈*E*, and *M* labels for the graph edges are assumed. The labels are enumerated from 0 to *M*−1. Let us take *a*∈{0,…,*M*−1}. The notation (*i*,*j*)=*a*states that the label of the edge connecting nodes *i* and *j* is equal to *a*. We also assume that the graphs are fully connected in the following sense. A graph can always be formally extended to a multi-labeled, fully connected graph by defining an edge label 0, the usual designation for no connection. For example, in binary graphs, which are the main subject of this article, (*i*,*j*)=1 means that nodes *i* and *j* are connected and (*i*,*j*)=0stands for a pair of disconnected nodes.

For each node *i*∈*V* we define the probability distribution *P*_*i*_(*a*), which is the fraction of nodes connected to node *i* by edges labeled *a*. In other words, if we choose a particular *i* and then randomly select another node, *j*, from the remaining *N*−1 nodes, the value of *P*_*i*_(*a*) is the probability of (*i*,*j*)=*a*. In a binary graph, *P*_*i*_(1) is the number of nodes connected to node *i* divided by *N*−1.

If we select two nodes, *i* and *j*, and randomly choose a third node *k*, *P*_*ij*_(*a*,*b*) is the probability that (*i*,*k*)=*a* and (*j*,*k*)=*b*. For example, to calculate *P*_*ij*_(1,0)in a binary graph, we count the number of nodes connected to *i* and not connected to *j* and divide it by *N*−2. The notation *P*_*ij*_(*a*,·) and *P*_*ij*_(·,*b*) stands for marginalization of *P*_*ij*_(*a*,*b*)over *j* and *i* respectively, i.e.,

(1) $$P_{\text{ij}}(a,\cdot )=\overset{M-1}{\underset{b=0}{\sum }}P_{\text{ij}}(a,b)\;\text{and}\;P_{\text{ij}}(\cdot ,b)=\overset{M-1}{\underset{a=0}{\sum }}P_{\text{ij}}(a,b).$$

Finally, conditional probabilities are defined as:

(2) $$P_{\text{ij}}(a|b)=\frac{P_{\text{ij}}(a,b)}{P_{\text{ij}}(\cdot ,b)}.$$

**Remark 1.***P*_*i*_(*a*)and *P*_*ij*_(*a*,·)are two probability distributions of random variables defined on the same alphabet {0,…,*M*−1}. The difference between these two quantities is small: both tend to zero as *N* goes to infinity. *P*_*i*_(*a*)describes a situation when only one node is selected, and we randomly choose another node. *P*_*ij*_(*a*,·)describes a situation when we are given a pair of nodes and a third node is chosen at random. The value of the random variable is the label of the edge between *i* and the selected node.

Shannon’s entropy will be denoted by *H*[·], e.g.,

(3) $$H[P_{i}(a)]=-\frac{1}{\log M}\overset{M-1}{\underset{a=0}{\sum }}P_{i}(a)\log P_{i}(a).$$

All logarithms in this article are to the base two. To normalize, the entropies are multiplied by 1/log*M*. Note that although the values of *H*[*P*_*i*_(*a*)] and *H*[*P*_*ij*_(*a*,·)] are normalized to the interval [0,1], the value of *H*[*P*_*ij*_(*a*,*b*)] is normalized to [0,2], since the maximal value of the entropy of a joint distribution is equal to the sum of entropies of the single variables. We want to preserve this property of entropies after normalization. The notation of *H*[*P*_*i*_(*a*)] and *H*[*P*_*ij*_(*a*,*b*)] will be abbreviated as *H*_*i*_and *H*_*ij*_, respectively. The set-complexity of a graph *G* is defined as

(4) $$\Psi (G)=C\overset{N}{\underset{i=2}{\sum }}\overset{i-1}{\underset{j=1}{\sum }}\max(H_{i},H_{j})m_{\text{ij}}(1-m_{\text{ij}}),$$

where *C* is a normalization factor of the form 8/(*N*(*N*−1))and *m*_*ij*_ is the normalized mutual information between nodes *i* and *j*,

(5) $$m_{\text{ij}}=\frac{1}{\log M}\overset{M-1}{\underset{a,b=0}{\sum }}P_{\text{ij}}(a,b)\log\frac{P_{\text{ij}}(a,b)}{P_{\text{ij}}(a,\cdot )P_{\text{ij}}(\cdot ,b)}.$$

We previously introduced the definition of mutual information for graphs [1]. Intuitively, it measures the reduction of the uncertainty about the connectivity of one node given the connectivity pattern of a second node. It is therefore natural to define this quantity as mutual information between random variables described by distributions *P*_*ij*_(*a*,·) and *P*_*ij*_(·,*b*), c.f., Remark 1.

In the remainder of the article we will be exploiting a useful fact [6] that *m*_*ij*_ can be rewritten as

(6) $$m_{\text{ij}}=H[P_{\text{ij}}(a,\cdot )]+C_{1}\overset{M-1}{\underset{a,b=0}{\sum }}P_{\text{ij}}(a,b)\log P_{\text{ij}}(a|b),$$

where *C*_1_=1/log*M*. Consequently,

(7) $$m_{\text{ij}}=H[P_{\text{ij}}(a,\cdot )]+H[P_{\text{ij}}(\cdot ,b)]-H_{\text{ij}}.$$

## Complexity of CBGs

A set of nodes in a CBG can be represented as a sum of two disjoint sets *O*_1_ and *O*_2_ such that if nodes *i* and *j* belong to different sets, then (*i*,*j*)=1, and if they belong to the same set, then (*i*,*j*)=0. Sets *O*_1_ and *O*_2_are referred to as *orbits*. This is consistent with the graph theory definition of an orbit, which holds that an orbit is an equivalence class of nodes under the action of an automorphism [7]. This means that all nodes in an orbit are connected in the same way to other nodes. The symbol *K*_*m*,*N*−*m*_ is used to denote a CBG of size *N*, where *m* is the size of *O*_1_.

Consider nodes *i* and *j* from the same orbit. By the definition of CBGs, (*i*,*k*)=(*k*,*j*) for any third node *k*. Thus, *P*_*ij*_(0,1)=*P*_*ij*_(1,0)=0. Consequently, *P*_*ij*_(0,0)=*P*_*ij*_(0,·)=*P*_*ij*_(·,0)and *P*_*ij*_(1,1)=*P*_*ij*_(1,·)=*P*_*ij*_(·,1). This leads us to *P*_*ij*_(0∣0)=*P*_*ij*_(1∣1)=1 and *P*_*ij*_(0∣1)=*P*_*ij*_(1∣0)=0. Similar reasoning holds for nodes from different orbits such that *P*_*ij*_(*a*∣*a*)=0and *P*_*ij*_(*a*∣*b*)=1 for *a*≠*b*. If we apply this result to Equation (6), we can see that the second component of the sum on the right hand side of the equation is zero. Therefore, we have proved the following lemma.

**Lemma 1.** If *G* is a CBG, then for any pair of nodes *i* and *j*

$$m_{\text{ij}}=H[P_{\text{ij}}(a,\cdot )].$$

Next we elucidate the relationship between entropy and the set-complexity of CBGs. However, we first have to deal with the difference between *H*_*i*_and *H*[*P*_*i*_(*a*,·)]. This problem can be resolved by introducing a common approximation for these two entropies. This is doable, because the difference between *P*_*ij*_(*a*,·)and *P*_*i*_(*a*)converges to zero with the increasing size of the graph, c.f., Remark 1. Let us show a common approximation of these entropies in the case of binary graphs. Suppose nodes *i* and *j* are in *O*_1_, then *P*_*i*_(0)=(*m*−1)/(*N*−1), *P*_*ij*_(0,·)=*P*_*ij*_(0,0)=(*m*−2)/(*N*−2). Both values can be reasonably approximated by *m*/*N* for large *N*. A similar analysis reveals that *P*_*ij*_(1,·)and *P*_*i*_(1) can be approximated by (*N*−*m*)/*N*. Thus, the common approximation for *H*_*i*_and *H*[*P*_*ij*_(*a*,·)]should have the following form:

(8) H(q)=−qlogq−(1−q)log(1−q),

where *q* = *m*/*N*. A similar analysis shows that Equation (8) can also be used to approximate entropies when *i*,*j*∈*O*_2_ or *i*∈*O*_1_, *j*∈*O*_2_. The notation *H*(*q*)emphasizes that this quantity depends only on the proportion of nodes in orbits *O*_1_ and *O*_2_ and does not depend on the size of the graph.

**Theorem 1.** Let *G*_*N*_be a sequence of complete bipartite graphs, such that the ratio *q*=*m*/*N*is constant for all *N*. Then, $\underset{N\to \infty }{\lim}\Psi (G_{N})=4(H^{2}(q)-H^{3}(q))$.

**Proof.** Lemma 1 allows us to rewrite each component of the sum on the right hand side of Equation (4) as

(9) $$\max(H_{i},H_{j})H[P_{\text{ij}}(a,\cdot )](1-H[P_{\text{ij}}(a,\cdot )]).$$

□

Since the values of all entropies presented above converge to *H*(*q*), it holds that

(10) $$\underset{N\to \infty }{\lim}\Psi (G_{N})=C\overset{N}{\underset{i=2}{\sum }}\overset{i-1}{\underset{j=1}{\sum }}(H^{2}(q)-H^{3}(q)).$$

Note that the sum on the right hand side of Equation (10) consists of *N*(*N*−1)/2 identical elements. Thus, Equation (10) can be rewritten to the equation of the theorem. QED.

**Figure 1:**
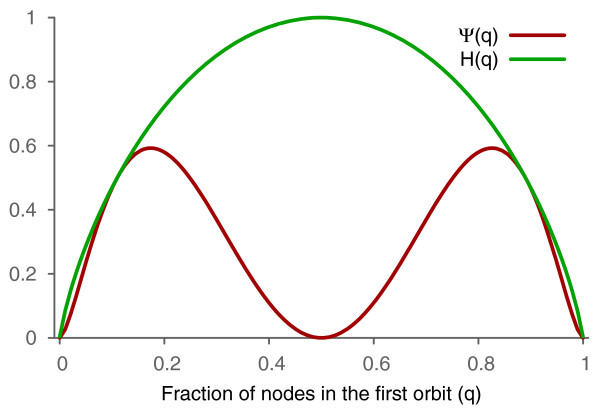
**Set-complexity and entropy versus sizes of orbits.** The set-complexity of CBGs (red) and their entropy (green) as functions of the proportion of nodes in one orbit to the other.

We see that the complexity of CBGs depends only on the entropy (mutual information is fully expressible in terms of the entropy for CBGs); thus, the complexity depends only on the sizes of *O*_1_ and *O*_2_. The equation in Theorem 1 is maximized for *H*(*q*)=2/3 leading to *Ψ*_max_≈0.59, the highest obtainable value of *Ψ* for CBGs. The complexity of CBGs is maximized when *q*≈0.174, i.e., when one of two orbits contains about 17.4*%*of nodes, see Figure 1. On the other hand, if both orbits are equal, entropies are close to one (more formally, they tend to one, when *N* goes to infinity), and the value of the set-complexity is close to zero.

We can easily see that the complexity can be bounded:

(11) $$\Psi (G)\leq \frac{2}{N(N-1)}\overset{N}{\underset{i=2}{\sum }}\overset{i-1}{\underset{j=1}{\sum }}\max(H_{i},H_{j}).$$

Figure 1 shows that CBGs with low values of *q* have complexity that is very close to the upper bound. Complexity of CBGs with high node entropies tends to zero (as the upper bound raises at the same time). This suggests a method for construction of complex graphs from CBGs.

## Complex binary graphs

At the end of the last section we show that the graphs with high values of *Ψ*(close to one) should exhibit high values of node entropies similar to *K*_*N*/2,*N*/2_ graphs. This section shows that, even though *K*_*N*/2,*N*/2_graphs have zero complexity, they are a good starting point for constructing highly complex graphs, in that a relatively small number of modifications is needed to increase *Ψ* substantially. We propose a stochastic transformation *F*_*p*_of a graph such that for any pair of nodes *i* and *j* the label of (*i*,*j*) is flipped to the opposite value with a probability *p*. We use *G*^∗^to denote the graph produced by this transformation applied to *G*.

Let us consider a sequence of graphs *G*_*N*_. We have seen already that in this case the non-zero joint probabilities converge to 0.5 when *N* tends to infinity. Therefore, the entropies *H*_*i*_and mutual information *m*_*ij*_converge to one, which implies

(12) $$\underset{N\to \infty }{\lim}\Psi (G_{N})=0.$$

Let us apply the transformation *F*_*p*_to *G*_*N*_. We want to describe the complexity of the sequence of transformed graphs $G_{N}^{*}$. To illustrate the impact of the transformation on the joint probabilities *P*_*ij*_(*a*,*b*), take nodes *i*, *j*, and *k* from the same orbit, so that (*i*,*k*)=(*j*,*k*)=0. The probability that labels of both edges will be flipped to one is equal to *p*^2^, the probability that only one label will be flipped is 2*p*(1−*p*), and the probability that both labels will not be flipped is (1−*p*)^2^. Thus, if for the original graph *P*_*ij*_(*a*,*a*)≈0.5(i.e., nodes *i* and *j* are from the same orbit as in the example above), then after the transformation we expect that the probabilities *P*_*ij*_(*a*,*b*)(for *a*≠*b*) will be equal to *p*(1−*p*), and the probabilities *P*_*ij*_(*a*,*a*)will become 1/2−*p*(1−*p*), or more formally

(13) $$\begin{array}{l} E[P_{\text{ij}}(0,0)]=E[P_{\text{ij}}(1,1)]=\frac{1}{2}-p(1-p),\\ E[P_{\text{ij}}(1,0)]=E[P_{\text{ij}}(0,1)]=p(1-p), \end{array}$$

where *E*[·]stands for the expected value. A similar analysis conducted for nodes from different orbits reveals that *E*[*P*_*ij*_(*a*,*a*)]=*p*(1−*p*) and *E*[*P*_*ij*_(*a*,*b*)]=1/2−*p*(1−*p*), where *a*≠*b*.

We see that the expected value of the node entropies remains one, i.e., the transformation preserves the entropy of nodes in *K*_*N*/2,*N*/2_ graphs, but it alters the mutual information *m*_*ij*_. The complexity is maximized when *m*_*ij*_=1/2. Since the node entropies are close to one, it follows from Equation (7) that *m*_*ij*_=1/2 when *H*_*ij*_=3/2. We can calculate that for the transformation *F*_*p*_, *E*[*H*_*ij*_]=3/2 iff *p*≈0.058428. This discussion can be summarized in the following theorem.

**Theorem 2.** Let *G*_*N*_be a sequence of graphs, and let $G_{N}^{*}$ be a sequence of corresponding outputs of the transformation *F*_*p*_with *p*≈0.058428. Then, $\underset{N\to \infty }{\lim}E[\Psi (G_{N}^{*})]=1$.

**Figure 2:**
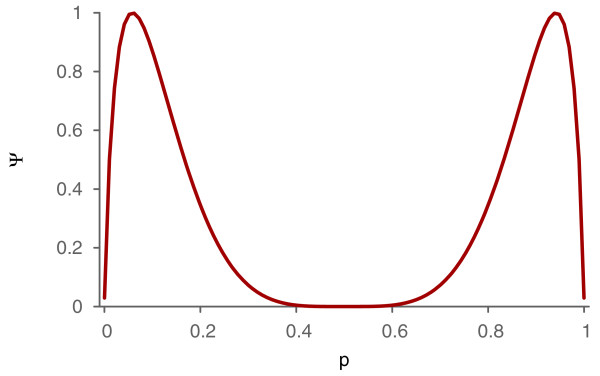
**Dependency of the set-complexity on the flip probability.** The relation between the probability, *p*, that an edge of a CBG will be flipped and the set-complexity, *Ψ*, of the CBG.

This argument demonstrates that if we apply the transformation *F*_*p*_ to large graphs, the outcome will be a graph with the complexity close to one. Since the relative number of transformed edges is low, the bipartite structure of the graph is largely preserved. The relation between the probability of transforming an edge and the expected value of the set-complexity of a CBG is plotted in Figure 2. In this figure, we can see that the complexity grows rapidly for small values of the probability. The decrease after the maximum can be inferred from the fact increased randomness beyond this point decreases the mutual information between nodes severely, degrading the set-complexity.

To illustrate this theorem experimentally, we applied the transformation to *K*_*N*/2,*N*/2_ graphs with *N*=50, 100, 200, 300, 500 nodes. The average values of *Ψ*(*G*^∗^)ranged from 0.9154 (with standard deviation 0.0185on 500 experiments) for *N*=50to 0.9926 (with standard deviation 0.0004 on 50 experiments) for *N*=500.

## Applications

We have shown how to construct high *Ψ*graphs. The method used for this construction enforces modular structure of these graphs. One may ask whether modularity is a property of all complex graphs. The answer, for these binary graphs, is yes. In [8], we proved that any complex graph must exhibit a modular structure; however, there can be some “noise” in the structure. The nature of that noise is similar to that described above. In other words, a binary graph with the set-complexity score close to one exhibits a structure similar to an outcome of the transformation *F*_*p*_ applied to *K*_*N*/2,*N*/2_ with *p*≈0.058. This result was generalized to graphs with *M*>2, so-called multi-colored graphs. For this generalization we extended the definition of a CBG, and defined complete multi-partite graphs (see [8] for more details). In the same article we analyzed some examples of complex graphs (*Ψ* of these graphs was close to one). To describe the information content of these graphs we used a histogram of

(14) $$\phi_{\text{ij}}=\max(H_{i},H_{j})m_{\text{ij}}(1-m_{\text{ij}}).$$

It is obvious that *Ψ* can be expressed as the average of *ϕ*_*ij*_.

One way of extending the analysis of a graph may be described as a problem similar to retrieving a signal from a noisy transmission of information. Here, the signal is a sub-graph showing some type of regular structure, e.g., a set of nodes with similar connectivity pattern, and the noise comes from all the nodes that do not exhibit any regular connectivity patterns, such as the nodes of a random graph. Structures like this arise in biology whenever we locate members of a large set of objects based on some common properties, for example, when we select genes based on their correlated expression levels. In contrast to [8], we focus our attention on graphs with very low values of the complexity score. Low complexity graphs can have different characters: some of them may be simple random graphs, while others can have a very regular structure, like CBGs. Both of these types of graphs are uncommon in biological applications. On one hand, biological systems are not random; thus, characteristics of their network representations cannot exhibit values similar to those of randomly generated graphs. On the other hand, such graphs are not completely regular. In biological sciences we almost always deal with an interesting mixture of randomness and regularity. We will focus our attention here on graphs whose structure is a mix of random and regular connectivity patterns.

Generally speaking the proposed approach focuses on finding a specific subset of nodes, a sub-graph, with a high contribution to *Ψ*. In order to do this we construct a histogram of *ϕ*_*ij*_, the complexity score for a specific pair of nodes, and for every node *i* define the following quantity:

(15) $$\Phi_{i}(T)=\overset{N}{\underset{j=1}{\sum }}\langle \phi_{\text{ij}}>T\rangle ,$$

where *T* is a threshold for values of *ϕ*_*ij*_and 〈·〉 stands for the Iverson’s bracket, i.e., a logic function that takes value 1, if the statement inside the bracket is true, and 0 otherwise. In summary, for a specific *i*, *Φ*_*i*_(*T*) is the number of pairs (*i*,*j*) in the graph such that *ϕ*_*ij*_>*T*. By looking at the rightmost tail of the histogram of *Φ*_*i*_(*T*) we can identify nodes with the highest contribution to *Ψ*.

We now present two examples. The first one is an artificially generated graph and the second is based on a biological data set. The two examples are followed by the discussion of the proposed approach: relation to community detection, modularity of networks/data sets, possible applications and plans for future work. We want to stress that the purpose of this discussion is to show that the set-complexity, and its components *ϕ*_*ij*_, of a graph gives us an insight into the graph’s structural properties. Nevertheless, this approach may also be interesting for analyzing real biological data.

### Example 1: artificially generated graph

In the first example we use a 300 node graph consisting of two sub-graphs. The first sub-graph is a *K*_25,25_ graph and the second is a random graph (also randomly connected to the CBG) in which the probability that two nodes are connected is 1/2. The probability of an edge between a pair of nodes from different sub-graphs is 1/2. Another example, based on real biological data, is given in the second example.

The graph overall exhibits a very low value of *Ψ*, relative to most CBGs, about 0.011. Low complexity indicates, in this case, a graph with a high number of randomly connected nodes. On the other hand, a low *Ψ* graph can be characteristic of a very regular graph structure. Looking at mutual information simply allows us to distinguish between a very regular and a very random graph. In the present example mutual information is low: its mean value is about 0.02. At the same time all node entropies are close to one. This indicates that the structure of the graph is more random than regular. Nevertheless, there is a modular sub-graph in this graph.

**Figure 3:**
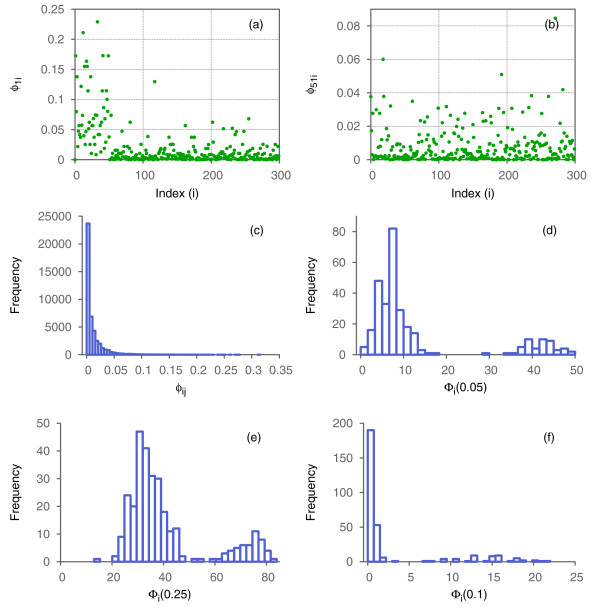
**Complexity analysis of the artificially generated graph.** Plots of **(a)***ϕ*_1*j*_and **(b)***ϕ*_51*j*_. **(c)** Histogram of *ϕ*_*ij*_for all pairs of nodes in the graph. Histograms of *Φ*_*i*_(*T*)for **(d)***T*=0.05, **(e)***T*=0.025, and **(f)***T*=0.1.

There are 299 *ϕ*_*ij*_values for every node *i* in the graph (number of undirected pairs that include *i*). The values of *ϕ*_*ij*_ for nodes within the CBG sub-graph should be, on average, higher than for nodes from a random sub-graph. Figure 3a,b show values of *ϕ*_1*j*_ and *ϕ*_51*j*_ respectively. We can see that for *i*=51most of *ϕ*_*ij*_ values are lower than 0.05, while for *i*=1, a considerable fraction of pairs (*i*,*j*)have *ϕ*_*ij*_>0.05(most of these belong to the complete bipartite sub-graph).

Figure 3c shows the histogram of *ϕ*_*ij*_. As expected, most of these values are concentrated close to zero, and the right tail is almost invisible. Nevertheless, the right tail is present, and the comparison of *ϕ*_*ij*_for *i*=1 and *i*=51indicates that nodes from the complete bipartite sub-graph make stronger contributions to the tail than nodes from the random sub-graph. To illustrate this we fixed the threshold, *T*=0.05, and calculated the number of pairs with *ϕ*_*ij*_>0.05, defined as *Φ*_*i*_(0.05). Figure 3d shows the histogram of *Φ*_*i*_(0.05).

**Figure 4:**
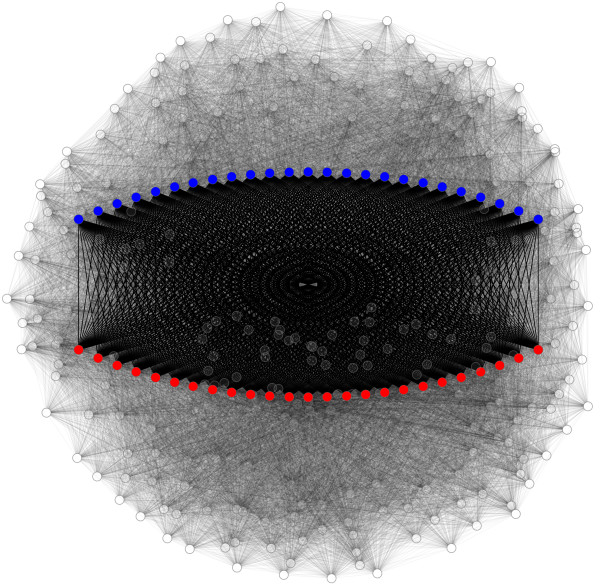
**Capturing the regular structure in the artificially generated graph.** This graph (disregarding the color) represents the binary graph from the first example of the article. The graph, which is mainly random, contains a complete bipartite sub-graph *K*_25,25_. This highly structured sub-graph, highlighted in the figure, was successfully detected by the set-complexity-based approach. The two modules of the *K*_25,25_graph are shown in red and blue.

Two groups of nodes are clearly distinguishable. The nodes in the right component of the histogram (*Φ*_*i*_(0.05)>25) are the 50 nodes of the complete bipartite sub-graph, whereas the nodes in the left component are from the random sub-graph. Figure 4 illustrates the entire graph and highlights the detected component.

Let us take a closer look at what happens when we change *T*. Figure 3e,f show histograms of *Φ*_*i*_(0.025) and *Φ*_*i*_(0.1), respectively. The complete bipartite sub-graph can be identified in both cases; however, in the first case (*T*=0.025) both groups of nodes are close to one another. Decreasing *T* below 0.025will result in misclassification of a significant number of nodes (mixing the two classes clearly separable in the present case). On the other hand, increasing *T* makes the group on the right more flat, therefore it becomes more difficult to distinguish between these groups. For example, in Figure 3f we show the histogram of *Φ*_*i*_(0.1) where the right group looks almost like a long tail of the group on the left.

As we can see the choice of the threshold *T* can be somewhat arbitrary at the outset. Our approach yields a tool for analyzing graphs. Thus, it could be used in a supervised mode, where *T* is specified by the user, or the threshold could be systematically scanned in an unsupervised mode.

### Example 2: biological data set

**Figure 5:**
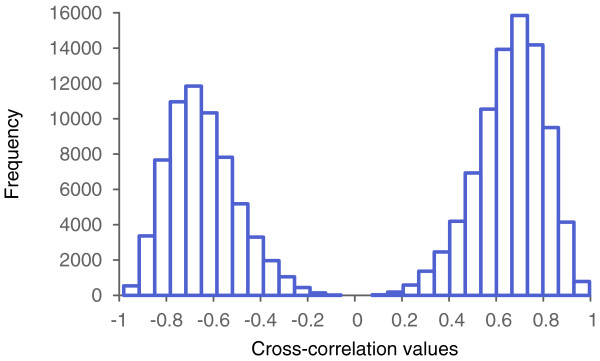
**The data from the biological example.** Histogram of cross-correlations between time series of expression levels of 547 genes during the cell cycle of the HeLa cells.

The second example is based on a real biological data set, and is both more realistic and more difficult. The data is a set of cross-correlations between time series of expression levels of 547 genes showing periodic variations during the cell cycle of the HeLa cells. These correlations were computed from data presented in [9]. Figure 5 shows a histogram of this data set. We represented this data as a network with three types of edges (I, II, III) corresponding to high positive correlation (>0.8) between two genes, high negative correlation (< −0.8), and intermediate-to-no correlation (between −0.8and 0.8). Up to now we have considered only binary graphs. This example, however, requires a ternary graph, *M*=3.

We want to solve the problem of finding a set, or sets, of nodes with a similar connectivity pattern, which might represent a modular sub-graph. This case is more difficult, because we do not know *a priori* that there is any modular structure. Consequently, we initially choose low values for *T*, to avoid omitting potentially relevant nodes.

**Figure 6:**
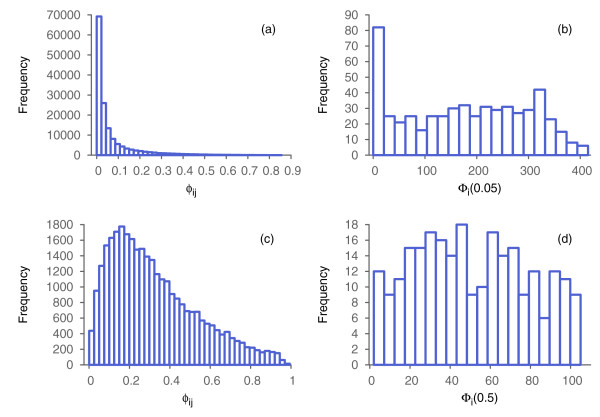
**Complexity analysis of the graph generated from the biological data.** Histograms of **(a)***ϕ*_*ij*_and **(b)***Φ*_*i*_(0.05)for the original graph. Histograms of **(c)***ϕ*_*ij*_and **(d)***Φ*_*i*_(0.5)for the selected 251 node sub-graph.

We start by analyzing the set-complexity and its components, *ϕ*_*ij*_. The value of *Ψ* is small: about 0.06. Figure 6a shows the histogram of *ϕ*_*ij*_for the correlation graph. The histogram is similar to the one discussed in the previous example (see Figure 3c). We set the threshold, *T*, to 0.05and compute *Φ*_*i*_(0.05)for all *i*, resulting in the histogram in Figure 6b. Note that this histogram is different from that presented in Figure 3d, where a subgroup of nodes is clearly separated from the others. We define a sub-graph of the original correlation graph by identifying only nodes for which *Φ*_*i*_(0.05)>200. We then redefine the graph as the sub-graph containing only these nodes, and then repeat the analysis. Since only nodes within the sub-graph are used in this calculation, the *ϕ*_*ij*_will, in general, all be different, and a new threshold will need to be set.

The new graph consists of 251 nodes. Recomputing *Ψ*and *ϕ*_*ij*_shows that the set-complexity of this graph is significantly higher—about 0.32. The histogram of the recalculated *ϕ*_*ij*_, presented in Figure 6c, is very different from the histogram in Figure 6a. We set a new threshold *T* equal to 0.5 and calculate the values of *Φ*_*i*_(0.5)for all *i*. The histogram of this is presented in Figure 6d. Selecting 97 nodes for which *Φ*_*i*_(0.5)>60 results in a graph whose complexity is about 0.7. It exhibits a bi-modular structure containing 33 and 64 nodes in each of the two modules. The nodes within each module are strongly connected via edges of type I (strong positive correlation), and nodes from different modules are usually connected via edges of type II (strong negative correlation). Some nodes are also connected via the type III edges. Table 1 shows the exact number of edges of different types within and between the modules. The genes present in the modules indicated in Table 1 are significantly enriched for genes known to be directly involved in the cell cycle. The detailed analysis of this network, however, its structure and biology, will be discussed in a future publication.

**Table 1:** Distribution of different types of edges in the 97 node sub-graph of the original correlation graph

	**Type I edges**	**Type II edges**	**Type III edges**
	**(strong positive correlation)**	**(strong negative correlation)**	
Module 1 (33 nodes)	**373 (70.6%)**	25 (4.7%)	130 (24.6%)
Module 2 (64 nodes)	**1644 (81.6%)**	46 (2.3%)	326 (16.2%)
Connections between modules	109 (5.2%)	**1405 (66.5%)**	598 (28.3%)

In parentheses we show the percentage of edges of each type in the set of all edges for each module as well as between the modules. In bold we indicate the largest edge type for each graph component.

## Conclusion

We have shown that, in general, a modular structure maximizes the set-complexity of a graph. It has been formally proved, however, that this is not always the case. If a binary graph is composed of two modules of identically connected nodes (orbits) and the modules have the same sizes, then the complexity of such a graph is almost zero. The complexity grows rapidly, however, when we perturb the graph structure by breaking this symmetry. The symmetry can be broken in two ways: either the number of nodes in the components of the CBG can be made unequal, or the complete bipartite character can be broken by adding or deleting edges [8]. Actually, the number of altered edges that can significantly increase *Ψ* is a relatively small number; and the bi-modular structure of the graph is essentially preserved in a graph with significant *Ψ*. Similar results can be obtained for multi-colored edge graphs, with *M*>2[8]. We presented a method and two examples here that suggest useful applications of the described theory to analyzing real biological data—finding highly informative modular sub-graphs in a large graph.

There are several technical aspects of the analysis presented above that need to be considered. First, in the second example, the procedure was applied iteratively, twice. We chose a sub-graph of interest and repeated the procedure on this sub-graph. It is important to note that in the iterations the values of *ϕ*_*ij*_ were recomputed for the sub-graph only: the nodes and edges that are not in the sub-graph are omitted from computation. Since the set-complexity is defined as a context dependent measure, we treat one subset of nodes as a context for the other subset. Therefore, by omitting a group of nodes we change the context for the remaining nodes and change the complexity. It is clear that the subset of nodes considered is an important part of the definition of the set-complexity.

Our examples illustrate how to use set-complexity to capture the information content of a graph. For instance, histograms on Figure 6a,c show the increase of information when we narrow the original graph from 541 to 251 nodes. This information gain is also quantified by the set-complexity, which increases from 0.06 to 0.32. This can be useful for an evaluation of a network. Even if a network seems to be uninformative, we can attempt to extract an informative set of hidden regular patterns by narrowing down the set of nodes. This can be especially useful for networks with multiple types of edges (multi-color graphs), for which existing community detection and clustering methods are not suitable.

We wish to point out a significant potential relationship between two ideas presented here. The notion of modularity, based on the common connectedness of sets of nodes, as reflected in the measure of mutual information in the graph, is closely related to the idea of redundancy. This is because the modularity often stems from sets of nodes that are connected in similar ways to other nodes. Redundancy, in turn, has a strong functional significance in all functional systems, which is that it provides a robustness against damage or loss. If there are two or more nodes that are connected in almost the same fashion, loss of one of these nodes or its connection(s) can be mitigated to some extent by having a stand in, or partial stand in, in another node. Clearly this is a quantitative issue that needs more attention to fully characterize. What is also clear is that with too much redundancy, or regularity, the range of responses and the sensitivity to a variety of inputs is limited. This qualitative notion parallels the very idea of maximizing *Ψ* in that regularity (similar to redundancy) is balanced against variety (similar to randomness). The idea is appealing in thinking about biology, in that the robustness to perturbation or damage and the sensitivity to perturbation of damage are two general properties that biological evolution seeks to balance in many ways. It may be that *Ψ* can provide some quantitative insight into this biological balancing act.

Though the concept of set-complexity, defining a balance between regularity and randomness, is promising for future applications in biology, the two examples in this article are illustrations of a possible approach based on set-complexity and should be viewed as complementary to traditional community detection algorithms. At the current stage of development, the proposed approach requires supervision, but it is clear that scanning through threshold parameter space will be a key to automating the method. Since this article (as well as [8]) provides a rigorous theoretical background for the set-complexity of graphs, it should be possible to derive an automated approach for performing an analysis as illustrated in the examples. One possible direction for future research is to combine the search for a maximally complex sub-graph with optimization techniques, such as hill-climbing, using stochastic sampling methods.

Another interesting extension to our work is to look at how to use set-complexity as a specific measure of the modularity of graphs and of data sets. This extension would allow us to analyze modularity of multi-labeled graphs, which is currently impossible using traditional measures of modularity, since there is no defined interpretation of modularity for graphs with various types of labels. This will be a direction for future work.

The set-complexity was originally defined as a measure of complexity of sets of binary strings [1]. This definition can easily be used for characterizing the complexity of dynamics of various types of Boolean networks (for example, random, probabilistic), in which a binary string represents a state of a network and, thus, a dynamic trajectory of a network is a set of strings [1,10]. We have defined the set-complexity in terms of Kolmogorov complexity [1]. Unfortunately, since Kolmogorov complexity is incomputable, it needs to be approximated by algorithmic compression of binary strings, which represent states of the network. This approach has two drawbacks: (1) the approximated set-complexity is not normalized, so it is difficult to compare complexities of networks with different size, and (2) we can say nothing about the structure of the sequences: we can only hypothesize that these strings should be somewhat similar to one another but, in contrast to the graph case, we cannot quantify these relations. It may be interesting to calculate the complexity of a set of strings in a manner similar to that presented in the current article. We have begun this type of analysis, and the preliminary results look promising. We believe that such an approach may give us interesting insights into the dynamics and information structures of various types of Boolean networks.

We have demonstrated that the probabilistic description of the set-complexity sets up a formal framework for reasoning about some properties of our measure of complexity. We are able to prove some important properties of the set-complexity of graphs. Such an approach can be fruitful in the further investigations of this subject. This may result in better understanding of the nature of complexity in system biology, which may play a key role from the perspective of practical applications of that theory.

## Abbreviations

CBG: complete bipartite graph.

## Competing interests

The authors declare that they have no competing interests.

## References

[B1] Galas DJ Nykter M Carter GW Price ND Shmulevich I Biological information as set-based complexity IEEE Trans. Inf. Theory 2010 56 667 677 10.1109/TIT.2009.2037046 PMC5110148 27857450

[B2] Kolmogorov AN Three approaches to the definition of the concept quantity of information (Russian) Probl. Peredachi Inf 1965 1 3 11

[B3] Carter GW Galas DJ Galitski T Maximal extraction of biological information from genetic interaction data PLOS Comput. Biol 2009 54 e1000347 19343223 10.1371/journal.pcbi.1000347 PMC2659753

[B4] Carter GW Rush CG Uygun F Sakhanenko NA Galas DJ Galitski T A systems-biology approach to modular genetic complexity Chaos 2010 20 026102 10.1063/1.3455183 20590331 PMC2909309

[B5] Ignac TM Sakhanenko NA Galas DJ Koeppl H, Acimovic J, Kesseli J, Maki-Marttunen T, Larjo A, Yli-Harja O Relation between the set-complexity of a graph and its structure Proceedings of the Eighth International Workshop on Computational Systems Biology: 6–8 June 2011 2011 Zurich, Switzerland: Tampere University of Technology, TICSP Series 81 84

[B6] Cover TM Thomas JA Elements of Information Theory 1991 New York: Wiley-Interscience

[B7] Gross J Yellen J Graph Theory and its Applications 1999 Boca Raton: CRC Press Inc

[B8] Ignac TM Sakhanenko NA Galas DJ Complexity of networks II: the set complexity of edge-colored graphs Complexity 2012 17 23 36

[B9] Whitfield ML Sherlock G Saldanha AJ Murray JI Ball CA Alexander KE Matese JC Perou CM Hurt MM Brown PO Botstein D Identification of genes periodically expressed in the human cell cycle and their expression in tumors Mol. Biol. Cell 2002 13 1977 2000 10.1091/mbc.02-02-0030. 12058064 PMC117619

[B10] Maki-Marttunen T Kesseli J Kauffman S Yli-Harja O Nykter M Koeppl H, Acimovic J, Kesseli J, Maki-Marttunen T, Larjo A, Yli-Harja O On the complexity of Boolean network state trajectories Proceedings of the Eighth International Workshop on Computational Systems Biology 2011 Zurich, Switzerland, 6–8 June 2011: Tampere University of Technology, TICSP Series 137 140

